# Role of HIV Subtype Diversity in the Development of Resistance to Antiviral Drugs

**DOI:** 10.3390/v2112493

**Published:** 2010-11-11

**Authors:** Mark A. Wainberg, Bluma G. Brenner

**Affiliations:** McGill University AIDS Centre, Jewish General Hospital, 3755 Cote-Ste-Catherine Road, Montreal, Quebec, H3T 1E2, Canada; E-Mail: bluma.brenner@mcgill.ca

**Keywords:** HIV, drug resistance, subtypes, reverse transcriptase, protease, integrase

## Abstract

Despite the fact that over 90% of HIV-1 infected people worldwide harbor non-subtype B variants of HIV-1, knowledge of resistance mutations in non-B HIV-1 and their clinical relevance is limited. Due to historical delays in access to antiretroviral therapy (ART) on a worldwide basis, the vast majority of reports on drug resistance deal with subtype B infections in developed countries. However, both enzymatic and virological data support the concept that naturally occurring polymorphisms among different nonB subtypes can affect HIV-1 susceptibility to antiretroviral drugs (ARVs), the magnitude of resistance conferred by major mutations, and the propensity to acquire some resistance mutations. Tools need to be optimized to assure accurate measurements of drug susceptibility of non-B subtypes. Furthermore, there is a need to recognize that each subtype may have a distinct resistance profile and that differences in resistance pathways may also impact on cross-resistance and the selection of second-line regimens. It will be essential to pay attention to newer drug combinations in well designed long-term longitudinal studies involving patients infected by viruses of different subtypes.

## 1. Introduction

Most HIV infections worldwide are due to non-subtype B infections [1]. HIV-1 group M has been classified into subtypes, circulating and unique recombinant forms (CRF and URF, respectively) because of its significant natural genetic variation. These include subtypes A–D, F–H, and J–K and many CRFs and URFs. Subtype B is the most prevalent in the Western World (Western Europe, the Americas, Japan and Australia), whilst non-B subtypes predominate in the rest of the world: subtype C in sub-Saharan Africa India, and Brazil, CRF01_AE in South East Asia, CRF02_AG in West Africa, and subtype A in Eastern Europe and Northern Asia [1–3]. In addition, the proportion of non-B subtypes in North and South America and Western Europe is increasing [4–7]. Combination antiretroviral therapy (ART) is now used in many areas of the world and HIV resistance to antiretroviral drugs (ARVs) has emerged in all locales. Thus, it is expected that non-B subtypes will become even more common in western countries.

Diminished sensitivity to ARVs in non-B subtypes has been less well studied than in subtype B, mainly because of the predominance of subtype B in those countries in which ARVs first became available, coupled with the availability of genotypic and phenotypic antiretroviral drug resistance testing [8]. Yet, there is a potential for genetic differences among subtypes to yield differential patterns of resistance-conferring mutations in response to ARV pressure. This possibility is supported by the fact that HIV-1 naturally varies in genetic content by as much as 35% among subtypes. Such variation is higher in some areas of the genome (40% in the env gene) and lower in others (8–10% in the pol, gag and IN genes) [8]. Because differences in codon sequences at positions associated with drug resistance mutations might predispose viral isolates from different subtypes to encode different amino acid substitutions, it is possible that HIV-1 genetic diversity may influence the type of resistance mutations that might eventually emerge upon drug exposure as well as the rate of emergence of resistance [8–9]. This diversity may also affect the degree of cross-resistance to ARVs of the same class, with the potential to impact on clinical outcomes, preservation of immunological responsiveness, and virologic failure [8].

One of the first examples of this was data from studies of single dose nevirapine (sdNVP) for prevention of mother-to-child transmission (PMTCT). Clinical trials demonstrated a disparity in the overall resistance among subtypes, with frequencies of 69, 36, 19 and 21% against nevirapine in women with subtype C, D, A, and CRF02_AG infections, respectively, despite the absence of pre-treatment resistance [10–13]. Ultrasensitive PCR detection procedures, which reveal minority species resistance, have revealed a higher incidence of nevirapine resistance (K103N, Y181C) in 70–87% of individuals with subtype C, compared with 42% of individuals with subtype A [14–16].

Similarly, evaluations of virological and biochemical data suggest that natural amino acid background can affect the magnitude of resistance conferred by many antiretroviral resistance mutations [17]. This is best illustrated by HIV-2 and group O viruses that show high-level innate resistance to non-nucleoside reverse transcriptase inhibitors (NNRTIs) through resistance mutations that are present as natural polymorphisms (Table 1) [18–19]. In contrast, many studies on antiretroviral drug resistance in non-B subtypes exposed to chronic suppressive therapy have yielded less definitive results with respect to the importance of natural HIV-1 diversity as a factor leading to differences in types of drug resistance mutations and the propensity to develop drug resistance in the first place.

Genotypic ARV resistance testing is of proven benefit in deciding on best choice of ARVs for individual treatment and serves as a repository of information on HIV resistance mutations. Several factors underscore the difficulties in defining inter-subtype differences. While genotyping can classify the major viral subtypes, significant proportions (∼15%) of infections remain unassigned or differentially assigned using different subtyping algorithms [8,20–21]. Although HIV resistance databases make efforts to incorporate newer subtype data into their pools of data, the availability of HIV genotypes from areas of the world with non-B subtype predominance is comparatively low [22], due to less availability of ARV therapy, the high cost of drug resistance testing and limited opportunities for research in resource-limited areas. Furthermore, resistance tests may often be performed only on participants enrolled in study cohorts or trials but not in general practice.

## 2. Results and Discussion

### 2.1. Resistance to Nucleoside Reverse Transcriptase Inhibitors (NRTIs)

In Botswana, subtype C patients treated with ZDV/ddI developed an atypical thymidine analogue resistance pathway (67N/70R/215Y) compared to subtype B [23], that was also not observed in patients with subtype C in India, South Africa, or Malawi [24–27]. Another study from Botswana reported a high incidence of K65R (30%) in subtype C patients who received d4T/ddI plus NVP or EFV [28]. In a much larger study, results from Malawi detected K65R or K70E in 23% of patients failing first line therapy with d4T/3TC/NVP [26], while K65R was detected in 7% and 15% of patients in South Africa failing first or second line regimens, respectively, whose nucleoside backbones included d4T/3TC or ddI/ZDV [29–30]. In addition, a study from Israel reported a high frequency of K65R in subtype C viruses from Ethiopian immigrants [31]. Finally, a recent report from India has shown that K65R was present in about 10–12% of patients who had received d4T/3TC/NVP in first-line therapy [32]. Differences in K65R and thymidine analogue mutations (TAMs) could be ascribed to treatment regimen and disease stage [24–27]. Access to viral load testing lead in India was associated with early detection of NNRTI-treatment failure, preventing acquisition of TAMs and K65R [24]. Other studies support regional differences among subtype C sub-epidemics from Ethiopia, Brazil and sub Saharan Africa, that impact on the RT backbone and NRTI resistance rates [8,33–34].

**Figure 1.**
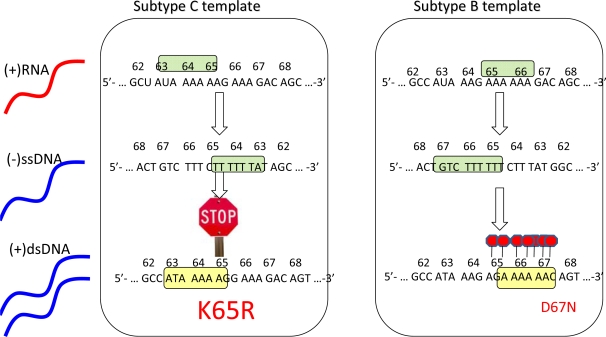
Schematic depiction of the template-based propensity of subtype C *versus* B viruses to develop the K65R mutation.

The presence of higher rates of the K65R mutation in subtype C [26,28–29] suggests that these viruses may have a particular predisposition toward acquiring this mutation, as has been described *in vitro* [35]. Importantly, a subtype C RNA template mechanism has been proposed to explain this phenomenon that involves higher rates of K65R mutagenesis in subtype C viruses than in other subtypes (Figure 1) [36–37]. Moreover, this mechanism is strictly template dependent and is independent of the source of the RT employed [36]. In subtype C, there is an intrinsic difficulty in synthesizing pol-A homopolymeric sequences that leads to template pausing at codon 65, facilitating the acquisition of K65R under selective drug pressure [37–38]. In contrast, the subtype B template favors pausing at codon 67 that may facilitate the generation of D67N and TAMs rather than K65R pathways [37–39].

Advanced ultra-deep pyrosequencing (UDPS) techniques have been used to detect the spread of K65R as transmitted and/or minority species in treatment-naïve populations [40–41]. Patients harboring subtype C infections showed a higher frequency of K65R than subtype B variants (1.04% *vs.* 0.25%) by UDPS but these differences were not replicated using limiting dilution clonal sequencing approaches [40]. The discrepant UDPS findings are consistent with PCR-induced pausing, leading to low-level spontaneous generation of K65R in subtype C. This does not, however, negate the higher risk of development of K65R in subtype C populations failing regimens containing d4T, ddI, or tenofovir (TFV) [32]. In addition, development of K65R in subtype C and CRF01_AE has been associated with the Y181C nevirapine mutation within the viral backbone [30,42].

Drug resistance selection studies showed that subtype C selected the K65R mutation faster under TFV pressure compared to subtype B [35]. However, K65R may be less frequent in subtype A than in all other subtypes [43]. A higher propensity to acquire TAMs was reported in patients carrying CRF_06 (AGK recombinants) as compared to patients carrying CRF02_AG from Burkina Faso [44].

In conclusion, the differential selection of K65R pathways in subtype C is related to template differences, ddI and d4T-containing regimens, as well as Y181C in the viral backbone. Thymidine analogue pathways are favored with zidovudine-based regimens. More extensive genotypic studies are required to ascertain subtype differences in acquisition of resistance to NRTIs. Figure 1 illustrates the basis for the preferred selection of K65R in subtype C.

### 2.2. Resistance to Non-nucleoside Reverse Transcriptase Inhibitors (NNRTIs)

Tissue culture selection studies have shown that a V106M mutation commonly develops in subtype C viruses following drug pressure with NVP or EFV, unlike the V106A mutation that is more commonly selected in subtype B. The basis for this difference is a nucleotide polymorphism at codon 106 in reverse transcriptase (RT) [45–46]. The clinical importance of the V106M mutation in non-B subtypes has been confirmed in recent years with six studies showing that V106M is frequently seen in non-B subtypes (C and CRF01_AE) after therapy with EFV or NVP [23,25,27,47–50].

The G190A mutation was also relatively more frequent among subtype C infected patients failing NNRTI-based therapy in Israel and India. In the Israeli but not the Indian study, G190A/S was seen as a natural polymorphism in subtype C from Ethiopian immigrants [25,49]. In both studies, the frequencies of these mutations among treated patients were higher than in subtype B and C drug-naïve patients.

While the overall prevalence of V106M in subtype C is higher than subtype B (12% *vs.* 0%) in individuals failing NNRTI-based regimens, K103N (29% *vs.* 40%) and Y181C (12% *vs.* 23%) remain important pathways for both subtype C and B, respectively [51]. There appear to be only minor differences in HIV resistance pathways in subtypes A, B, and C with the second generation NNRTI, etravirine [50].

### 2.3. PR Mutations

With respect to PR, the D30N mutation was not observed in CRF02_AG and CRF02_AE isolates from patients failing NFV therapy; rather, the N88S mutation emerged after NFV use in CRF01_AE and after IDV use in subtype B [52–53]. A third study reported an absence of the D30N mutation in CRF01_AE, but no information on the specific type of PIs received by the patients was provided [54]. A low frequency of D30N was seen in subtype C isolates from Ethiopian immigrants to Israel after NFV usage *versus* a higher frequency in subtype C viruses from Botswana [55–56], suggesting that subtype C viruses from Ethiopia (the origin of the samples identified in Israel) and southern Africa might behave differently. The M89I/V mutations have been observed in F, G and C subtypes but not in other subtypes [26]. The V82I natural polymorphism in subtype G led to the emergence of I82M/T/S with treatment failure [57]. Finally, the L90M mutation is rare in subtype F but common in subtype B from Brazil [58]. A recent paper suggests that polymorphisms at position 36 in PR may play important roles in determining the emergence of specific patterns of resistance mutations among viruses of different subtypes [59].

Molecular dynamic simulations were performed to gain an understanding of the underlying mechanisms leading to the overall higher preponderance of D30N in subtype B relative to other subtypes. D30N appears to selectively confer resistance to NFV in subtype B by increasing the flexibility of the protease (PR) flap region and destabilizing the PR-inhibitor complex [60]. For subtype C, D30N required the accessory N83T mutation to confer resistance and rescue fitness [61].

Two comprehensive surveys have reported differences in natural protease polymorphisms among non-B subtypes [62–63]. In these studies, positions less frequently mutated in non-B subtypes than in subtype B after exposure to ARVs included changes at PR residues 10, 20, and 63 in subtype A; residues 20, 53, 63, 74, and 82 in subtype C; residues 13 and 20 in subtype D; residues 10, 14, 20, and 77 in subtype F; residues 20, 67, 73, 82, and 88 in subtype G; residues 20, 63, 82, and 89 in CRF01_AE; and residue 20 in CRF02_AG [63].

One study revealed higher rates of accumulation of NRTI and PI resistance mutations and equal rates of emergence of NNRTI mutations in subtype B compared to C [64]. An observational study from southern Brazil also showed a lower relative frequency of primary resistance to PIs in subtype C as compared to subtype B and is suggestive that PI mutations may be less well tolerated at the structural level in the former subtype [65].

In general, HIV-1 subtype diversity has not limited the overall benefit of ART (Table 1). However, there are subtype differences in the type and preference of pathways of resistance with some mutations emerging almost exclusively in some non-B subtypes, e.g., the protease mutations 82M in subtype G *versus* 82A/F/S in the others, 88D in subtype B *versus* 88S in subtypes C and CRF02_AG [66]. HIV-2 has major mutations in regard to NRTIs, NNRTIs, and PIs, which contribute to innate NNRTI resistance and rapid development of multi-class drug resistance (Table 1) [67–68]. The RT mutation V106M in subtypes C and A *vs.* V106A in subtype B is observed with NVP and EFV. Polymorphisms at RT residue 98, common in subtype G, are associated with NNRTI resistance in subtype B and may lower the resistance barrier and duration of efficacy of NNRTIs [69]. The available evidence indicates that the frequency of some resistance mutations shared by B and non-B subtypes can vary after failure of first line therapeutic regimens, as in the case of the K65R mutation; these differences in type and frequency of resistance mutations should not be underestimated. The TAM pathway 67N/70R/215Y found in subtype C in Botswana will probably be adequately detected by most resistance algorithms, since it does not involve new mutations.

One study reported a lower risk for accumulation of major (primary) resistance mutations in subtype C than B [64]. Qualitatively, the major mutations that emerged in both subtypes were the same. The fact that both subtypes B and C patients had similar profiles of virological failure after use of the same ART regimens rules out ancillary factors responsible for the difference. Several minor mutations in subtype B PR may appear as frequent natural polymorphisms in several non-B subtypes (e.g., M36I, L89M) [58–59]. The L89M polymorphism can lead to the M89I mutation that confers resistance to PIs. Thus, it makes sense that there might be a lower accumulation of major mutations in C subtypes, if we assume that these natural polymorphisms act similarly in subtype C as they do when present as secondary resistance mutations in subtype B.

Overall, the majority of non-B HIV-1 subtype isolates possess wild-type susceptibilities similar to those of subtype B wild-type isolates. Compared to B subtypes, diminished susceptibilities among wild-type isolates have been found for CRF02_AG recombinant viruses in three different studies in regard to NFV and ATV [63,69–70]. However, no study assigned statistical significance of drug susceptibility levels due to polymorphisms and small sample size. One study performed molecular modeling and suggested that distortions in the K26 pocket of A/G proteases appear to be responsible for a lower binding energy of NFV and hence lower susceptibility of A/G viruses to this drug [70]. Others have also found A/G isolates with lower susceptibilities to certain PIs (NFV and ATV). Only one study has detected an important proportion of WT isolates with lower susceptibilities to ATV [71]. Most phenotypes have been determined by commercial or in-house assays that were developed primarily to measure B-subtype drug susceptibilities based on the laboratory adapted strains HXB2 or NL43. These studies employed a modified clone of a laboratory strain that lacks both the terminal part of Gag and most of Pol in order to generate a recombinant clone.

Thermodynamic studies performed on target-inhibitor interactions in PR have specifically described a lower affinity of non-B subtype proteases for PIs and amplification of primary resistance mutations on the basis of background polymorphisms.

Few data on the potential for cross-resistance to PIs among non-B subtypes have been published. In regard to NFV, there is a tendency to select for the L90M pathway in subtype C instead of D30N. Competition assays appear to support a lower fitness of subtype C viruses bearing D30N, which could explain the above findings [61].

### 2.4. Implications for Clinical Practice

Resistance in non- B subtypes has rarely been reported on the basis of single drugs or NRTI backbones but, rather, mutations have been reported for specific drug classes. Hence, cross-resistance can be estimated only for some NRTIs and NNRTIs but not for most PIs that are the only drugs eligible as part of second-line regimens in most regions of the world. For NFV, the potential for cross-resistance in viruses of CRF01_AE and CRF02_AG origin could be higher than has been observed in subtype B, due to the preferential selection of the N88S and L90M mutations. Data of this sort are not yet available for most PIs in the context of non-subtype B viruses. Also, NRTI backbones may also vary in the mutation profile they select for according to the drugs that are combined. Newer drugs (e.g., TDF and ATV/r) are now preferred both in resource-rich countries and non-B subtype prevalent populations. HIV resistance databases continue to enter HIV genotype data from non-B subtype variants, but so far few data sets are available (Stanford HIV resistance database, ANRS, *etc.*) for drugs that have become first line therapy in developed countries, e.g., tenofovir, atazanavir, darunavir, etravirine and raltegravir.

### 2.5. Future Research

The clinical and prognostic implications of the preferential emergence of some mutations and changes in the frequency of these mutations in select non-B subtypes need greater attention. Future research on the role of polymorphisms in non-subtype B viruses that increase in frequency after drug exposure and may contribute to drug resistance (e.g., A98G/S in RT and M36I and K20I in PR) [72] is merited. This could be particularly important in parts of Africa in which treatment failure has been reported in as many as 40% of patients after two years [73] and in India where resistance rates of 80% to two drug classes have been reported after failure on first-line regimens that employed NRTI/NNRTI combinations [74]. No study has yet tested the degree of resistance or cross-resistance that certain mutational combinations (67N/70R/215Y) may confer *in vitro*. Future studies should try to assess pre- and post-treatment genotypes in order to find associations of certain polymorphisms with drug resistance, including variations of polymorphisms in variants of the same subtype that are located in different geographical areas. This might improve the appropriateness of selection of certain drugs over others in the context of second or third line therapeutic options.

With few exceptions, the different studies conducted in populations affected by non-subtype B viruses are too heterogeneous to permit pooling of data [8]. These studies have addressed different research questions and used non-equivalent NRTI backbones (e.g., ZVD/ddI and ZDV/3TC). They have also grouped mutations by drug class without information on the nature of the regimen at virologic failure, and have reported resistance in different ways (e.g., different algorithms or resistance lists), making it difficult to relate resistance mutations to a specific drug or drug combination. In order to better recognize inter-subtype differences, more longitudinal studies on response to first-line ARV combinations are needed. Of course, pre- and post-therapy genotype resistance testing is also necessary.

## 3. Conclusions

Biochemical and virological data provide compelling evidence on the differential effect of genetic background on both the type and degree of antiretroviral drug resistance in HIV-1. In regard to HIV protease, genetic background can affect the degree of protein binding caused by primary mutations and restore the function of protease (PR) to a differential degree, according to the background polymorphisms in each subtype. This effect was not discernible in the absence of typical major resistance mutations, but rather when particular backgrounds of combinations of major resistance mutations and background polymorphisms were present. In this regard, some background polymorphisms can clearly act as secondary resistance mutations.

HIV phenotypic assays have failed to find differences of large magnitude in the susceptibilities of B *versus* non-B subtypes. This is consistent with what has been observed at a molecular level. However, there are few data on relative susceptibility levels among subtypes carrying specific major resistance mutations. More information is required, because many polymorphisms in non-B viruses are considered to be secondary resistance mutations based on the fact that they emerge in B subtype viruses after drug exposure. However, the effect of such polymorphisms within different genetic backgrounds cannot always be extrapolated to non-B subtypes. They might sometimes contribute to higher levels of resistance in certain genetic backgrounds but could also have either a neutral effect or hypersensitize HIV to ARVs, e.g., I93L is a secondary resistance mutation in subtype B but causes hypersusceptibility to PIs in subtype C [61].

Novel NNRTI resistance mutations have been found in subtype C that had not been recognized in subtype B. In tissue culture, subtype C can acquire a different mutation (the V106M substitution) under NNRTI drug pressure compared to what is seen in subtype B, and this mutation confers broad cross-resistance to an extent that supersedes the equivalent V106A mutation subtype B.

Rates of acquisition of resistance could have important implications in regard to durability of therapy. *In vitro*, the emergence of the K65R mutation is faster in subtype C than in B [30,35]. Biochemical mechanisms have been proposed to explain this observation in subtype C templates processed by the RT polymerase [36–38,75]. In the clinic, K65R has been seen in approximately 70% of patients failing ddI-containing nucleoside backbones in Botswana [28]. However, K65R does not appear to emerge frequently in subtype C patients who have received either TDF or TDF/FTC as part of a triple therapy regimen [30]. This is doubtless a reflection of the use of well-tolerated effective drugs that have long mutually reinforcing intracellular half-lives that act in combination to suppress viral replication and prevent the emergence of resistance mutations. However, higher numbers of patients and longer follow-up will be required to assess if there is a consistent impact of subtype C in the emergence of K65R in clinical settings.

Multiple clinical and *in vitro* studies have confirmed that protease and gag are a functional unit and co-evolve when HIV is subject to drug pressure. Both clearly mutate under PI pressure and Gag mutations can act as compensatory substitutions that may increase levels of resistance and viral replication capacity. None of the recombinant phenotyping systems used for clinical samples monitors Gag. The magnitude of differences among Gag may vary between 2 to 2.5 fold between subtypes. Although this difference appears small, different subtypes might develop compensatory Gag mutations at different rates, establishing a need to take Gag into account in phenotyping. Only one study reported that a recombinant construct included Gag of clinical origin, but did not test the same subtypes as others.

A wide variety of mutations can impact on sensitivity to differential extent. In contrast, such information cannot yet be generated with regard to non-B subtypes due to a scarcity of paired genotypic and phenotypic data. Only three studies analyzed genotypes and phenotypes of non-B subtypes in clinical trials: one on use of single dose NVP for prevention of mother-to-child transmission and two clinical trials of double and triple NRTI combinations that are now obsolete [8].

The potential of cross-resistance acquires importance in settings with limited access to antiretroviral therapy. Few *in vitro* comparative data are available for protease inhibitors in non-B subtypes, yet such data may be crucial to understanding cross resistance to specific drugs [58–59]. Importantly, PIs may be the only potentially widely accessible option for drug sequencing in salvage therapy in most resource-limited settings. Moreover, the fact that resistance to PIs commonly requires that large numbers of resistance mutations be present may result in a situation in which the individual contribution of any single mutation to drug resistance, with some exceptions will be slight. This is a definite advantage of using drugs with a high genetic barrier for the development of drug resistance. It also follows that differences among subtypes with regard to development of drug resistance are more likely to be important for NRTIs and NNRTIs than for PIs.

Large numbers of paired samples need to be systematically collected from naïve and treated patients infected with subtypes C, AE, AG, A and G, toward genotypic and phenotypic analysis for both established drug classes as well as for newer classes such as integrase inhibitors.

## Figures and Tables

**Table 1.** Subtype signature polymorphisms and mutations in reverse transcriptase (RT) or protease (PR) that may impact on emergent resistance to nucleoside and non-nucleoside reverse transcriptase inhibitors (NRTIs and NNRTIs) and protease inhibitors (PIs), respectively.

**Drug Class**	**Subtype**	**Polymorphism or Mutation**	**Drug**	**Mutation(s)**	**Reference**
**Reverse Transcriptase**					

**NRTIS**	C	64–65–66 KKK motif T69N, V75I, V118I, L210N, T215S, K219N	ddI, d4T, TDF	K65R	[30]
	HIV-2		NRTIs	TAMs/ K65R	[66]

**NNRTIS**	C	V106V	EFV, NVP	V106M	[45]
	G	A98S	NNRTIs		[67]
	HIV-2	Y181I, Y188L, G190A, K101A, V106I, V179I	All NNRTIs	Cross-NNRTI resistance	[66]
	O	Y181C, A98S, K103R, V179E	All NNRTIs	Cross-NNRTI resistance	[18]

**Protease**					

PIs	Non-B	M36I	PIs		[58]
	G, AE	K20I	PIs		[62]
	G	V82I	PIs	I82M/T/S	[62]
	A,C,F,G, AE,AG	L89M	PIs	L89I	[69]
	HIV-2	L10I/V,K 20V, V32I, M36I, M46I, I47V, L63E/K, A71V, G73A, V77T, V82IL	PIs	APV and PIs	[66]

ddI, didanosine; d4T, stavudine; TFV, tenofovir; EFV, efavirenz; NVP, nevirapine
